# Party Politics vs. Grievance Politics: Competing Modes of Representative Democracy

**DOI:** 10.1007/s12115-022-00686-z

**Published:** 2022-03-14

**Authors:** Matthew Flinders, Markus Hinterleitner

**Affiliations:** 1 Department of Politics, University of Sheffield, Sheffield, UK; 2 LMU Munich, Geschwister Scholl Institute of Political Science, Munich, Germany

**Keywords:** Representative democracy, Crisis, Grievances, Blame, Political parties, Politicians

## Abstract

As a vast literature on political disaffection, populism, “pitchfork politics,” and the emergence of an “age of anger” testifies, the nature of democratic politics and the socio-political context in which it operates appear to have shifted sharply during the last decade. This is reflected in the rise of challenger parties, the election of unorthodox politicians, and widespread concern regarding the “crisis,” “death,” or “end” of democracy. Existing analyses have, however, understandably adopted a conventional model of party-based representative politics as their main interpretive lens or reference point to make sense of these changes. This article adopts a far bolder position. It suggests that a new form of “grievance politics” has emerged that constitutes a distinct and novel species of representative democracy. Grievance politics is defined by the fuelling and funneling of negative emotions and various blame-based political strategies which explicitly challenge and confound many of the core principles and values that have traditionally underpinned conventional conceptions of party politics. It is the tension between party politics and grievance politics—and their contemporary co-existence as competing modes of political representation—which this article seeks to underline and through this, to develop a clearer understanding of possible futures for representative democracy.

As a vast literature on the “crisis” (e.g., Przeworski 2019), “end” (e.g., Runciman 2018), “suicide” (Goldberg 2018), or “death” (Levitsky and Ziblatt 2018) of democratic politics has underlined—not to mention a more recent wave of literature on democratic backsliding and the emergence of illiberal democracies in the context of Covid-19 (Kettemann and Lachmayer 2021)—democracies have in recent decades been subjected to a range of pressures. Concerns about the crisis of democracy are far from novel. The Trilateral Commission’s (1975) report on the topic, for example, was published nearly 50 years ago. However, the elections of politicians such as Donald Trump in the USA and Boris Johnson in the UK have arguably exacerbated and increased longstanding concerns about “the twilight of democracy” (Applebaum 2020). This is reflected in a contemporary seam of inter-disciplinary scholarship that has analyzed “anti-democratic tactics,” “sectarianizing behavior,” “disciplined messaging,” “moralized language,” “anti-establishment appeals,” “rhetoric of moral outrage,” and how politicians “tap into resentments” (see, for example, the work of Barr 2009; Cramer 2016; Finkel et al. 2020; Gentzkow et al. 2019; Lamont et al. 2017).

Politicians engaging in these behaviors have varyingly been labelled (authoritarian) populists, (xenophobic) nationalists, mavericks, illiberal, anti-politicians, or outsider-politicians. Scholars seem to agree that politicians such as Trump or Johnson offer an appealing counter project to established politicians and parties and their more conventional ways of “doing politics”—ways that have increasingly fallen out of favor with citizens (Foa and Mounk 2016; Norris and Inglehart 2018). In the past, public discontent with politics, democratic disaffection, and declining political trust were important drivers of party system evolution (Inglehart 2015). Changing public preferences were thought to induce a number of fairly predictable and well-understood changes on the supply-side of politics: from established parties adapting their orientations and party programs to new parties entering the political arena to the rise of new political forces such as social movements or “movement parties” (Hutter et al. 2019). For example, economic grievances (Kitschelt and McGann 1997), political elitism and corruption (Inglehart 1997), and immigration (Ivarsflaten 2008) help to explain the rise of the far right. Likewise, economic anxieties related to globalization and modernization, social envy, and a cultural backlash against the pluralization of societies help to account for the ascent of populism (Mudde and Rovira Kaltwasser 2018).

This article proposes a very different and distinctive argument. It suggests that the politics that recently culminated in the actions of politicians such as Trump or Johnson represents a distinct form of political representation and, by extension, a “new species” of representative democracy (O’Donell 1994). What we currently see is not simply the populist corruption of traditional ways of “doing politics,” but the emergence of distinct ways of addressing public preferences and injecting them into politics. We seek to articulate this change and to offer a new analytical framework that provides a comprehensive account of what is new and novel about our current politics and helps to better understand the emergence of democratic threats or pathologies.

This new form of political representation, which we term “grievance politics,” is decidedly different from classic representative democracy (or traditional conceptions of “party politics”) for at least three reasons. First and foremost, grievance politics is imbued with a fundamental sense of *negative civic energy*. Traditional politics revolves around—as Bernard Crick’s (1965) classic *In Defence of Politics* sought to promote and praise—a deep belief in the positive capacity of collective action to address societal problems and protect individuals from shared risks. In contrast, grievance politics revolves around the fueling, funneling, and flaming of negative emotions such as fear or anger. Grievance politics portrays problems or protests as the fault of a specific “other” (institutions, sections of community, etc.) and through this injects a divisive and polarizing force into politics. Secondly, in grievance politics, parties are no longer the *main agents of representation* but individual politicians are. Although the organizational forms of parties have changed over time (Katz and Mair 1995), scholars have long considered them to be the key linkage institution connecting citizens to politics. In grievance politics, individual politicians supplant parties as the dominant agents of representation and seek to nurture new forms of direct communication with mass audiences. Finally, the *strategies of representation* utilized by these politicians are fundamentally different from those of traditional political parties. Traditionally, parties articulate and aggregate public preferences in party programs, compete for votes and office, and seek to transform preferences into policy; but the new breed of politicians merely transforms citizens’ preferences into grievances and blame. As we will explain below, the adoption of a “populist style” (Moffitt 2016) is but one way in which grievance politicians “represent” public preferences. Moreover, and unlike parties, grievance politicians do not have a strong policy orientation and thus primarily offer symbolic representation to their constituents. Grievance politics is therefore often bound-up in very distinctive performative repertoires which deserve attention from those concerned with “the life and death of democracy” (Keane 2009).

In order to develop and substantiate the argument above, this article is divided into four inter-related parts. The first part presents a table that seeks to articulate the differences between traditional party politics and the emergence of a very distinct mode of grievance politics. This, we suggest, provides a novel analytical lens through which to develop a sharper and more accurate account of contemporary democratic change and challenge. The second part focuses on traditional party politics and its erosion, arguing that three common weaknesses of present-day democratic representation help to understand why grievance politics is on the rise in many advanced democracies. The third part of this article then zooms in on some of the key dimensions of grievance politics, showing that they are very different—almost diametrically opposed—to those of party-based models of democracy. By considering these key dimensions in their entirety and exposing their interrelations, it also becomes evident why grievance politics is not simply the populist corruption of party politics, but a distinct species of representative democracy. And yet the purity of “ideal types” is generally compromised by the messy realities of practical politics, which is why the final part of this article zooms in on the co-existence of party politics and grievance politics in actual democracies. It suggests that it is the competition between these two types—marked by either a shift towards grievance politics *within* party-based democratic landscapes or by the repression of grievance politics through the revitalization of party politics—that determines the future of representative democracy. The final part also hints at a number of factors and conditions which can be expected to influence the outcome of this competition. Note that this article is conceptual and solely draws on selective empirical examples from the two well-known grievance politicians Donald Trump and Boris Johnson. In doing so, we by no means suggest that grievance politics is constrained to the traditional political “right.” It is a defining element of grievance politics that political actors can engage in it independent of their possible political orientations. We therefore conclude by outlining avenues for more systematic and broader empirical research on this new species of representative democracy.

## Part I. Contrasting Ideal Types

The core argument of this article is that it is possible to identify the emergence of a new species of representative politics, one that needs to be acknowledged and analyzed in its own right and not solely as the corruption of traditional modes of “doing politics.” The aim of this section is to facilitate comparison between a classic conception of representative democracy (hereafter called “party politics,” or simply Mode I) and the emergence of what we suggest is a very different form of representative democracy (called “grievance politics,” or Mode II), both understood as ideal types (Goertz 2006). Table 1 provides an overview of both the parameters and core characteristics of party politics and grievance politics.

**Table 1:** Party politics and grievance politics

	Mode I: Party politics	Mode II: Grievance politics
Foundational Essence	Positive: belief in collective action against shared social risks	Negative: belief in emphasizing difference and stoking conflict
Mechanism of representation	Public preferences → party programs → policies	Public preferences → grievances → blame
Agents of representation	Political parties (as main linkage, connector, docking point, platform)	Individual politicians (populists, anti-politicians, mavericks, celebrities)
Strategies of representation	– Articulating and aggregating citizens’ preferences (through debate, deliberation, party programs, and ideologies) – Competing for votes/office – Policy-making	– Fueling grievances (i) by creating chaos and confusion, (ii) through fearmongering, and (iii) by accentuating tribal identities – Generating blame – Seeking blame
Constitutional emphasis	Rule-making	Rule-breaking
Emotional repertoire	Hope, belief, realism	Fear, anger, victimhood
Role of citizens	– Participation in politics through electoral choice – Open emphasis on both the rights and responsibilities of citizens	– Participation in “pitchfork politics” to express dissatisfaction – Citizens as an entertained audience watching forms of “celebrity politics”
Strengths	– Clear chain of delegation and broadly accepted “rules of the game” – Party politics demand the creation of “broad churches” either within parties or through coalitions – Aware of its own imperfections with attempts to “bolt on” deeper forms of public participation (e.g., “the deliberative turn”) – “Slow boring of hard woods” focuses on detail and restricts radical shifts	– Offers simple solutions to complex problems (i.e., the promise to “get things done”) – Ability to fuel and funnel social anxieties and utilize deep emotional triggers (e.g., the power of nostalgia) – Emphasis on rule-breaking, “bad manners” or sensationalist performative stunts guarantees media coverage – Makes “normal” politics look boring and bland
Weaknesses	– Parties may deny political representation to societal problems – Parties may fail to address societal problems through bold (and visible) policy action – Parties may weaken their role as agents of representation by delegating power to non-majoritarian bodies	– Grievance politicians do not have a strong policy orientation (and thus only symbolically represent citizens’ preferences) – Tendency to over-inflate the public’s expectations – May entrench collective action problems

The distinction presented between party politics and grievance politics in Table 1 forms the focal point and main contribution of this article. In seeking to highlight grievance politics as a fundamentally different mode of representative politics, we are working across a broad intellectual canvas that, in turn, demands the use of a fairly broad brush. Our hope is that by highlighting the existence and exceptionality of grievance politics we might provoke subsequent critique and analysis, thereby filling in the fine detail, texture, and tone.

And yet even at this early stage it is possible to highlight three strengths of the dichotomy offered in Table 1. First and foremost, it sets down both the parameters and core characteristics of what we argue is actually a new model, mode, or phase of democratic politics. This (secondly) allows us to demonstrate our core argument that grievance politics should not be studied or evaluated *through the lens* of traditional party politics for the simple reason that core characteristics of grievance politics then would unavoidably appear as little more than the corruption of party politics, a conclusion that will always and obviously generate negative evaluations. The foundational essence of both models is very different, and this finds form in various performative, process-based, and public-facing dimensions. The comprehensive consideration of these dimensions flows into a third and final point which concerns connecting different strands of scholarship on the crisis of democracy. In recent years, and as has already been mentioned, a huge amount of scholarship has been published that looks at the crisis of democracy at a very broad level (rising inequalities, cultural shifts, increasing apathy, the “populist zeitgeist,” etc.); at the same time, more specialized micro-political analyses have also been produced that raise focused concerns in relation to, for example, elite behavior, party membership, partisan dealignment, policy effectiveness, etc. And yet despite all of this research, the political and social sciences still lack a simple unifying narrative in response to current political transformations. We seek to provide this narrative through a focus on the emergence of grievance politics as a distinct but highly negative mode of representative democracy. In order to develop this argument, the next section looks very briefly at the decline of party politics.

## Part II. The Decline of Party Politics

Representative democracy, understood as an ideal type, rests on the notion that democracies have devised various ways in which public interests and preferences can be injected into politics (Pitkin 1972; Mansbridge 2011). Underlying this notion are “mechanisms of representation” in which political parties play an outsized role (Caramani 2017). Parties compete for citizens’ votes by articulating their preferences and aggregating them in party programs and ideologies. Through electoral competition, parties gain access to government power and subsequently transform citizens’ preferences into public policies. Parties’ strategies of representation constitute a “chain of responsiveness” that ideally connects citizens to politics and produces policies that citizens want (Powell 2004). Citizens accordingly participate in politics by voting for parties and their policy programs (based on rational and emotional considerations). Scholars have intensively debated the conditions for party politics to function consistently, which include “universal adult suffrage, free and competitive elections to choose policy makers, multiple information sources, multiple political parties, and civil and political rights” (Powell 2004, p. 91). While democracy in this conceptualization is clearly an ideal type that may never be attained in practice, after the end of World War II, many societies came very close to the ideal type on a number of important dimensions (Dahl 2020). With this in mind, it is useful to draw upon the existing research base (Caramani 2017; Wessels 2011; etc.) in order to identify three inter-related key weaknesses of present-day party politics: (i) *blaming citizens*; (ii) *lacking problem-solving capacity*; and (iii) *hollowing-out*.

Blaming citizens revolves around the reframing of individual-state relationships and is concerned with what Elizabeth Shove (2010) has labelled as “the ABC model.” In this model, social change is framed as a matter of the individual and the role of the state is restricted to shaping individuals’ “choice architecture” in order to “nudge” them into making “good” decisions. This rational-choice derived model of social action emphasizes personal responsibility and therefore dovetails with neo-liberal conceptions of the state. The role of politicians and public servants in this model is to “persuade, price, and advise” individuals on the basis that when given better information and appropriate incentives, individuals (i) will change their **a**ttitudes, (ii) alter their **b**ehavior, and/or (iii) make **c**hoices that are better aligned with addressing social challenges. As Keith Dowding (2020, p. xii) argues, “over the past 50 years, one specific ideological viewpoint has dominated. And that is the cult of personal responsibility.” What Dowding charts with great effect through a focus on gun crime, obesity, homelessness, gambling, and drugs policy in the USA and UK is either the gradual withdrawal of state-based control mechanisms (i.e., liberalization, regulation, etc.) or governments’ refusal to countenance the implementation of such measures. Whereas government once took on a degree of direct responsibility for the health and wealth of all their citizens, a process Dowding describes as “privatized blame-shifting” has occurred. Instead of recognizing citizens’ concerns as legitimate political claims, governments have placed responsibility for dealing with major social challenges on individuals—and this at exactly the same historical point that levels of social and economic inequality are increasing and forms of employment are more and more precarious.

Blaming citizens flows into a second weakness and the widespread perception that conventional party politics is for one reason or another unable to take bold decisions. Even in situations where parties do not shift responsibility to citizens but recognize the existence of major social challenges, they often struggle to make policy that effectively addresses them. Powerful veto forces, collective action problems, and inertia-inducing partisan polarization tend to frustrate the adoption of ambitious policies and combine to produce a well-documented risk-aversion within policy-making. Barack Obama’s (2020, p. 555) memoirs, for example, provide stark insights into the Herculean endeavor of trying to work through a gridlocked and labyrinthine governmental machine: “At times, I felt like the fisherman in Hemingway’s *The Old Man and the Sea*, sharks gnawing at my catch as I tried to tow it to shore.” Add to this the incentives created by a relatively short-term electoral cycle and the likelihood that long-term structural or systemic investments are likely to produce *distant effects* and the rationale for devolving responsibility to citizens through the “ABC model” becomes clear. The flipside, however, is that an increasing number of citizens appear to feel that government is simply less effective than it was in the past, and concerns about a lack of problem-solving capacity only increase as the public’s awareness of global challenges intensifies.

Against the backdrop of a lack of problem-solving capacity, politicians and scholars alike have called for the delegation of significant decision-making powers to scientific experts on the basis that as they are free from popular control, they are better able to take the tough decisions that need to be taken (e.g., Shearman and Smith 2007). Building problem-solving capacity through delegating power, however, brings us to our third weakness of party politics and a focus on “hollowing-out.” Instead of presenting themselves as the first contact point for public preferences, parties have increasingly downplayed their role as agents of representation by relinquishing actual policy-making power to “non-majoritarian” or “arm’s-length” bodies in order to depoliticize issues and provide a solution to the “credible commitment dilemma” (Majone 1997). This is the “unbundling” (Pollitt and Talbot 2014) or “unravelling” (Hooghe and Marks 2003) of the state that flows through Colin Hay’s (2007) *Why We Hate Politics*, Peter Mair’s (2013) *Ruling the Void*, and Paul Fawcett’s (2017) *Anti-Politics, Depoliticisation and Governance* and which highlights the narrowing of the sphere for which politicians are willing to accept *direct* responsibility. The point being made is simply that a model of representative politics based on the wholesale delegation of powers and responsibilities *away* from elected politicians is hardly likely to instill public confidence. If anything, this process is likely to fuel a “crisis of representation” and the generation of grievances. Taken together, blaming citizens, lacking problem-solving capacity and processes of “hollowing-out” are likely to create a situation where the “functioning of the representative linkages between parties and popular preferences is put into question” (Hutter et al. 2019, p. 329). The political opportunity structure shifts as a result, creating space for the emergence of a new kind of grievance politics.

## Part III: The Rise of Grievance Politics

Grievance politics has emerged as an analytically distinct form of political representation that is designed and intended to challenge and disrupt traditional party politics. As Table 1 illustrates, Mode II’s mechanism of representation is simpler than that of Mode I. It merely consists of the transformation of citizens’ (unaddressed) preferences into grievances and blame, and often involves the explicit criticism of Mode I institutions and processes. Grievance politics interprets the existence of political apathy or frustration vis-à-vis traditional politics as a potential political resource and therefore seeks to exploit and exaggerate the existence of social grievances *with* party politics. Its “anti-political” emphasis is therefore not “anti-politics” per se but is more accurately represented as being against or “anti” how party politics has evolved. The main agents of representation in Mode II are not political parties but individual politicians, which in the literature have varyingly been called (authoritarian) populists, mavericks, anti-establishment figures, or celebrity politicians (see also Barr 2009). Understanding Mode II’s mechanics of representation therefore requires us to focus on a different unit of analysis. We distinguish between three overarching strategies of representation employed by grievance politicians. All of them are primarily rhetorical strategies transported through classic media (mainly TV, but also newspapers) and social media (Twitter, Facebook, etc.): (i) *fueling grievances*, (ii) *generating blame*, and (iii) *seeking blame*.

In relation to fueling grievances, research in political psychology suggests that governments’ failure to address societal problems such as rising levels of economic inequality creates negative emotions like anger, fear, stress, or uncertainty among citizens. Citizens living in unequal societies, for example, “are significantly more likely to regularly experience negative, sanctioning moral emotions” (Hitlin and Harkness 2018; see also Wilkinson and Pickett 2010; Case and Deaton 2020). By fueling grievances, politicians can tap into these negative emotions, the logic being that appeals to unaddressed preferences activate (negative) emotions which in turn trigger (negative) political judgements (Brader 2005; Marcus 2000). Politicians can fuel grievances in at least three ways.

First, they can do so by *creating chaos and confusion*. As the Washington Post’s (2020) *Donald Trump and his Assault on Truth* illustrates in forensic detail, grievance politicians such as Trump have been shown to spread lies, fake news, conspiracies, disinformation, and misinformation (see also Bennett and Livingston 2018; Lazer et al. 2018). Peter Oborne’s (2021) *The Assault on Truth* explains how by creating chaos and confusion, grievance politicians not only reinforce negative emotions such as uncertainty or fear but also, and paradoxically, can gain authenticity. Lying, for example, is generally considered a flagrant violation of the norm of truth-telling. And yet in times of crisis, citizens tend to see norm violators as authentic champions of their preferences, who “can be perceived as bravely speaking a deep and otherwise suppressed truth” (Hahl et al. 2018, p. 3). This flows into a second way of fueling grievances—*fearmongering*. Grievance politicians can stir panic and fear in a wide variety of ways, which range from anti-immigrant claims (e.g., migrants destroying “our” culture) to election-fraud claims (e.g., “stop the steal”) to invoking threats of a financial panic or inflation (e.g., “you will lose what you have”). Fear appeals have two advantages for grievance politicians. First, as Brader (2005) notes, “[f]ear appeals – featuring content and imagery associated with threat – should motivate a search for information, decrease the salience of prior beliefs, and encourage reconsideration of choices on the basis of contemporary evaluations.” Moreover, threats are “experienced largely through affective channels rather than through explicit cognitive perceptions” (Marcus 2000, p. 232). Fear appeals thus help politicians to detach citizens from previous political and partisan allegiances and make them more vulnerable to the spread of chaos and confusion (see above). Fear appeals create a sense of vulnerability among people by emphasizing their “downward mobility” in society while simultaneously removing their own responsibility for it. As Lamont et al. (2017) observe, Trump “removed blame for [citizens’] downward mobility by pointing to globalization as a structural force.” Fear appeals can therefore offer a powerful counter-message to the “ABC model,” reframing what had been seen as private issues of individual choice as public concerns demanding collective responses.

Finally, politicians can fuel grievances by *accentuating tribal identities*. Research in political psychology suggests that people are group animals, and that this affects their (political) perceptions (Clark et al. 2019). Social identities and group attachments therefore figure crucially in peoples’ political perceptions and behavior (Achen and Bartels 2017; Cramer 2016). However, strong social boundaries do not necessarily coincide with negative feelings towards other groups but may simply be the result of strong in-group identification. This changes when grievance politicians manage to connect grievances perceived by some in-group to purported actions by some out-group (Leonardelli and Brewer 2001). Nationalist, nativist, sectarian, racialized, or welfare chauvinist claims, playing on resentments, or stirring social envy, therefore encourage citizens to think in “us-vs-them” terms. People who think in “us-vs-them” terms make their in-group feelings salient and simultaneously develop negative feelings and emotions for the out-group (Iyengar et al. 2012). Put differently, the accentuation of tribal identities through grievance tactics makes people engage in “negative boundary work,” i.e., they increasingly define and compare themselves to other social groups and are more aware of their own group’s maltreatment by others or its “rightful place in the national pecking order” (Lamont et al. 2017; Leonardelli and Brewer 2001). Donald Trump, for example, returned the white working class to prominence in American politics by addressing and portraying it as a “new minority” which had been ignored and silenced in national politics for decades (Gest 2016).

Political and social psychology research also suggests that various forms of fueling grievances are more successful if employed in combination rather than in isolation. Grievance politicians who convince people to be the members of a single, tightly delimited group or tribe lower their “social identity complexity,” i.e., their awareness of being members of multiple social groups. Low social identity complexity, in turn, is conducive to the development of prejudices towards others, and is likely to be present in situations where individuals exhibit a high need for certainty and stress reduction because of perceived threats (Brewer and Pierce 2005), situations that grievance politicians help create through fearmongering and the creation of chaos and confusion (see above). Overall, fueling grievances leads to abundant negative emotions among citizens.

This leads us into a second major dimension of grievance politics and to a focus on *generating blame*. Through the generation of blame, grievance politicians give citizens a target for their negative emotions. As Weaver (2018) argues, blame generation (or negative messaging) allows politicians to take advantage of the public’s loss aversion and tendency to privilege negative information. Mistrust of government in particular “provides fertile ground for more negative messages to be viewed as credible, and therefore be effective” (Weaver 2018, p. 275). Grievance politicians can direct blame at specific political rivals, or engage in a more populist variety of blame generation by blaming the political system, elites, the “deep state,” or the “D.C. swamp” (Moffitt 2016; Mudde and Rovira Kaltwasser 2018). By generating blame, grievance politicians convey the clear message that Mode I and its weaknesses are responsible for public grievances.

There is, however, a third blame-related dimension of grievance politics which serves to further illustrate the emergence of a distinctive mode of representative politics. Instead of routinely avoiding and deflecting blame like their conventional counterparts (Hinterleitner 2020), grievance politicians sometimes deliberately set out to *be blamed*. Breaking the rules, being provocative, rejecting etiquette, displaying bad manners, telling inappropriate jokes, repeating inaccurate statements, or threatening to break the law or constitutional conventions—“with ‘the middle finger’ defiantly raised” as Pierre Ostiguy (2017, 84) puts it, “to the well brought up, the proper, the accepted truths and ways associated with diverse world elites”—becomes a performative strategy for demonstrating difference and claiming authenticity. For example, throughout his initial campaign for the presidency and his time in office, Donald Trump repeatedly offended through the use of negative labels (e.g., “Crooked Hilary”) and through the outright denial of well-established facts and scientific consensus (e.g., unsubstantiated claims about COVID-19 cures). Boris Johnson is likewise known for a rather unconventional style which involved offending foreign dignitaries, a scruffy appearance, turning-up late, cronyism, the promotion of false statistics, and an inability to resist the temptation to make intemperate comments or jokes. By triggering retaliatory actions by established actors, “blame-seeking” becomes a method of almost trapping conventional politicians to demonstrate their allegiance to a model of politics that large sections of the public have lost faith with (Flinders, Hinterleitner, and Weaver, forthcoming). Moreover, blame-seeking provides grievance politicians with an opportunity to connect with those who feel “left behind” (Wuthnow 2019) or “strangers in their own land” (Hochschild 2016) as it triggers exactly those reactions by established actors that are widely associated with “distant” and “self-serving” elites.

Fueling grievances, blame-generating, and blame-seeking are strategies of representation that translate public preferences (or more specifically, concerns and anxieties) into negative and targeted emotions. Unlike political parties in Mode I, grievance politicians arguably offer a largely symbolic representation to citizens but tend to have very little interest or experience in policy delivery or the machinery of government. In fact, it is an important characteristic of grievance politicians that they do not have strong policy orientations. For example, observers have described Donald Trump and his administration as “post-policy,” i.e., devoid of a concrete governing agenda. Boris Johnson—as several biographers have noted—adopts a highly protean approach to policy. While grievance politicians routinely take positions on policy issues, they also frequently change them, are not particularly interested in their realization, and primarily use them to communicate values (Benen 2020). A paradoxical side effect of this post-policy orientation is that grievance politicians often leave their predecessors’ policies untouched. Donald Trump’s domestic policies, focusing on tax cuts and deregulation, did not significantly diverge from those of previous Republican presidents. Boris Johnson’s policies, too, are broadly in line with his Conservative predecessors.

As an ideal type, grievance politics is fundamentally different from traditional party politics as a mode of representative politics. Its foundational essence is negative, and it embraces a “divide-and-rule” logic which polarizes opinion and inflames fears. Grievance politics reduces the role of party platforms and increases the role of individual profiles; and it also highlights the changing emotional context within which political competition takes place. The relationship between the governors and the governed is recalibrated towards an emphasis on spectatorship and possibly even celebrity. This creates a clear link to the contemporary emergence of populism but a focus on grievance politics embraces a much wider range of variables and trends. Unlike populism, grievance politics does not constitute an unmediated form of rule (Caramani 2017). Instead, it is characterized by a new form of “mediators.” While these mediators may adopt a populist style (Moffitt 2016) to direct blame at Mode I, they also employ a host of other political strategies to fuel and funnel public grievances. Some of these strategies even run contrary to conventional populist claims. While populist claims are based on the distinction between a corrupt elite and a popular *majority* (Mudde and Rovira Kaltwasser 2018), the accentuation of tribal identities often portrays a particular group as a disregarded and mistreated *minority*. Mode II is thus not simply the populist corruption of Mode I, but a deeper and wider socio-political construct than populism. In fact, scholars who equate grievance politics with populism are unlikely to capture the interrelations and interactions that exist between populist claims on the one hand, and the many blame-based and emotion-related strategies that define grievance politics on the other. But considering grievance politics as a distinct form of political representation not only allows us to comprehensively capture how the actions of politicians such as Trump or Johnson transform democracies; a distinctive focus on grievance politics also provides us, we suggest, with a clearer idea of how representative democracy can be expected to change over time.

## Part IV: Futures for Representative Democracy

Almost exactly50 years ago, the Trilateral Commission published its landmark report—*The Crisis of Democracy* (Crozier et al. 1975)—which offered the first major comparative analysis of the rise of political apathy and democratic disengagement. Half a century later, the analysis of democracy remains largely framed in narratives of crisis, collapse, and catastrophe. It is in exactly this context that this article has attempted to make a bold and provocative argument that (i) critiques the existing research base for consistently approaching the analysis of democracy through the traditional lens of party politics (i.e., Mode I) and (ii) seeks to highlight the emergence of a completely new mode or model of representative democracy in the form of “grievance politics,” a model that explicitly challenges and confounds many of the core principles and values that have traditionally underpinned conventional party politics (i.e., Mode II). But what are the factors or conditions that frame the competition between party politics and grievance politics, and by extension, help to determine the future of representative democracies?

Whether existing democracies resemble more Mode I or Mode II (or show clear signs of hybridization between the two) will to some extent depend on a number of variables. First and foremost, *political polarization* and *macro-economic policy opportunities* are likely to define parties’ potential leeway as agents of representation. The more polarized a political system, the harder it is for parties to adopt effective policies in response to societal problems, as partisan polarization frequently leads to legislative gridlock (Barber and McCarty 2015). Moreover, citizens’ negative emotions in today’s democracies stem to a significant degree from repercussions of globalization such as disruptions in labor markets, pressure on salaries, and increased economic inequality (Lonergan and Blyth 2020). Several scholars have observed that democracies’ integration into the global economy creates an increasing gap between responsiveness to domestic demands and responsibility to international and supranational constraints that is exceedingly difficult to bridge for governments (Mair 2009; Schäfer and Streeck 2013). Therefore, the less capacity a country has vis-à-vis the global economy, the more impaired its parties’ leeway as agents of representation should be.

While polarization and limited macro-economic policy-making opportunities can be expected to create a deeper pool of unaddressed preferences, other factors should influence how effectively grievance politicians can exploit them, thereby also influencing their success prospects of turning grievance tactics into vote gains and electoral success. First, the *configuration of the media system*, especially the degree of fractionalization and the existence of informational “filter bubbles,” is relevant. Traditional media fulfil an important gatekeeping function in democracies by deciding on which political events to cover and how, by framing responsibility for political issues, and by exposing citizens to information that disconfirms their existing views (e.g., Iyengar 1990). This is relevant for the success prospects of grievance politicians, as media actors can decide whether to challenge the often simplistic claims of grievance politics, for example, by inhibiting the spreading of fake news or by discrediting excessive blame-seeking. Hence, the weaker the gatekeeping function of traditional media—notably in the sense of public service broadcasting—the higher the incentives for and success prospects of grievance politics should be. Second, the *gatekeeping function of traditional parties* is also likely to affect the political opportunity structure for grievance politicians. Gatekeepers, such as party elite “insiders” in the USA, tend to be risk averse in their preferences for presidential candidates. However, their ability to steer nominations towards mainstream candidates has been challenged for a variety of reasons (Cohen et al. 2016; MacWilliams 2016). In the UK, the “democratization” of party leadership processes in recent years has been directly linked to the selection of “outsider” candidates such as Jeremy Corbyn as Leader of the Labour Party and Boris Johnson for the Conservatives (Denham et al. 2020). Therefore, the weaker the gatekeeping function of political parties, the higher the probability that grievance politicians can capture and repurpose them.

Third, the existence of *identity-establishing political and social institutions* should influence the success prospects of grievance politics. Political and social institutions with stable political allegiances such as trade unions, churches, or associations of various kinds provide ground for citizens’ group membership and hence influence their political identities (Mair 2013; Putnam 2020). Such institutions should make it harder for grievance politicians to play on resentments and make tribal identities salient as they provide the social glue or bridging networks between specific groups. Social capital might therefore be seen as a bulwark against the emergence of crude grievance politics. Hence, the weaker and the less prevalent shared identity-establishing political and social institutions are in a democracy, the more receptive citizens are likely to be to the transformation of their negative emotions into grievances and blame. Finally, the success of grievance politicians should depend on *cultural, social, and political opportunities for fueling grievances*. For example, the peculiar isolationist tradition that exists in the USA provides ample opportunities for politicians to construct “foreign threats.” Likewise, the existence or visibility of social groups influences grievance politicians’ opportunities to accentuate tribal identities. For instance, Lamont et al. (2017, p. 155) show that “Trump capitalized on established boundaries in his appeal to workers, but also drew stronger boundaries toward undocumented immigrants, refugees, and Muslims, groups that gained salience in the last decades due to historical circumstances such as 9/11 and the Syrian civil war.” Equally important, the shift by many mainstream left-wing parties towards “cultural” issues parallel to watering down their traditional redistributive economic agendas helps to create the discursive space in which grievance politicians can make their sectarianizing claims. Many issues that preoccupy the contemporary mainstream left (e.g., gender pronouns) arguably are irrelevant to the lived experience of most working class voters and contribute to their alienation from Mode I party politics—a reality that provides grievance politicians with the possibility to portray mainstream politicians as out-of-touch with disaffected citizens. To summarize, existing democracies should increasingly resemble Mode II where the political system is polarized, macro-economic opportunities for bold policy action are limited, traditional media and parties struggle to perform their gatekeeping functions, and cultural, social, and political opportunities for grievance politics are widespread.

Based on these considerations, it is indeed plausible to project a one-directional trajectory in which democracies inexorably move away from Mode I and towards Mode II where several of the above-described factors enable important aspects of grievance politics. This trajectory is based on the assumption of a parasitic relationship where grievance politics undermines party politics by eroding the institutions that guarantee its functioning. For example, it is plausible to expect that the widespread fueling of grievances distorts public opinion and democratic debate, two factors that allow citizens to participate in politics through (informed) electoral choice. The distribution of fake news, for instance, creates political misperceptions among citizens (Nyhan and Reifler 2010). Likewise, the accentuation of tribal identities encourages citizens to “understand their circumstances as the fault of guilty and less deserving social groups, not as the product of broad social, economic, and political forces” (Cramer 2016, p. 21)—a development that makes it unlikely that citizens will vote for those parties that aim to address the true causes of societal problems through bold policy action. These arguments suggest that there is a tension between Mode I and Mode II, as the latter risks gradually chipping-away at the credibility of the former.

However, it is also plausible to predict the emergence of a countercurrent whereby citizens grow increasingly dissatisfied with grievance politics. In this alternative scenario, politicians seek to utilize the existence of deep-seated frustrations not to reject party politics but to remodel it towards a better-functioning version. This trajectory is based on the assumption that citizens eventually realize that Mode II represents a wholly negative and divisive brand of symbolic representation which is highly unlikely to deliver effective solutions to pressing social challenges. Broad-based political parties who promote the existence of choice and seek to re-engage as agents of representation may well flourish in such a context. While the above-described structural factors should importantly influence parties’ leeway in terms of representation, we also argued that their perceived unattractiveness in many existing democracies is partly self-inflicted. There is no law that dictates parties to deny political representation to societal problems and blame citizens for them, or to “hollow-out” Mode I’s representative linkages by relinquishing policy-making power to non-majoritarian bodies. Moreover, it is possible to imagine that innovative ways of “mending democracy” help to explicitly address public anger and social anxieties and transform them into new forms of bottom-up collective action (e.g., Hendriks et al. 2020; Salzman 2020). The future of democracy is therefore possibly more open and dynamic than many observers feel willing to acknowledge. The aim of this article has been to step back from specific frailties or dysfunctions of democracy and map the changing topography of citizen-elite linkages through a focus on two “ideal types” of representative democracy, thereby encouraging future debate and research in this space. We deem it particularly important for future research to zoom in further on grievance politics and its implications, tackling questions such as who is pursuing grievance politics and why, and how successful they have been in what kind of conditions.
